# The Prevalence of Toxoplasmosis in Bulgaria for the Period 2014–2023, with a Focus on Pregnant Women

**DOI:** 10.3390/pathogens14030270

**Published:** 2025-03-11

**Authors:** Iskra Rainova, Rumen Harizanov, Mihaela Videnova, Nina Tsvetkova, Raina Borisova, Eleonora Kaneva, Yana Todorova

**Affiliations:** 1 Department of Parasitology and Tropical Medicine, National Centre of Infectious and Parasitic Diseases (NCIPD), 26 Yanko Sakazov Blvd., 1504 Sofia, Bulgaria; rainova@ncipd.org (I.R.); mvidenova@ncipd.org (M.V.); tsvetkova@ncipd.org (N.T.); rainaborisova@ncipd.org (R.B.); kaneva@ncipd.org (E.K.); 2 Department of Immunology, National Centre of Infectious and Parasitic Diseases (NCIPD), 26 Yanko Sakazov Blvd., 1504 Sofia, Bulgaria; y_todorova@abv.bg

**Keywords:** *Toxoplasma gondii*, *Toxoplasma* antibodies, seroprevalence, pregnant women, toxoplasmosis

## Abstract

The specificity of the life cycle of *T. gondii*, the causative agent of toxoplasmosis, determines its relevance in some patient groups; especially in women of childbearing age. The objective of this study was to ascertain the prevalence of this parasitic infection in Bulgaria between 2014 and 2023, focusing on pregnant women and those applying for an in vitro procedure or after an abortion. During the period, 115,053 individuals were tested for toxoplasmosis in the country, with an average seropositivity rate of 16.96%. At the NRL for the Diagnosis of Parasitic Diseases, ELISA tests were utilized to ascertain specific *Toxoplasma* IgG, IgM, and IgA antibodies, IgG avidity, and PCR to substantiate the DNA of the parasite. Between 2014 and 2023, the laboratory conducted tests on 631 pregnant women, and 161 women applying for in vitro fertilization or after an abortion. In 24.7% of pregnant women, data demonstrated the presence of IgG antibodies only, and *Toxoplasma* IgG and IgM or IgG, IgM, and IgA antibodies were found in 16.6% and 8% of women, respectively. In the subset of individuals examined after an abortion or for an in vitro procedure, IgG was detected in 28%, IgG and IgM in 13%, and IgG, IgM, and IgA antibodies in 3.7%. Seroprevalence rises with increasing age, but does not show any dynamic compared to our previous studies. Our results show a higher seropositivity for toxoplasmosis in pregnant women in Bulgaria than in neighboring countries.

## 1. Introduction

Toxoplasmosis is a cosmopolitan protozoan disease. In immunocompetent individuals, it most often presents as asymptomatic or with self-limiting symptoms (e.g., lymphadenopathy, fever, and fatigue) during primary infection. However, in cases of congenital toxoplasmosis or immunocompromisation (particularly in individuals with AIDS), the disease can be severe and even life-threatening [1]. The causative agent of the infection is *Toxoplasma gondii*, a small intracellular protozoon belonging to the *Apicomplexa*. Humans and animals become infected through contact with domestic cats and other members of the *Felidae* family. Human and animal intermediate hosts are infected mainly by the dietary route, and less commonly by the aquatic route, through water, vegetables, green herbs, ground fruits, and hands contaminated with parasite oocysts, or by eating undercooked meat and meat products infected with tissue cysts [2]. Infection can also occur through organ transplantation or vertical transmission in acute, primary acquired toxoplasmosis in pregnant women. Primary infection with *T. gondii* during pregnancy can lead to congenital toxoplasmosis, which can have severe consequences for neonates and fetuses, including developmental delay, blindness, epilepsy, miscarriage, and stillbirth [3]. The prevalence of toxoplasmosis varies between different regions of the world. The highest prevalences have been reported in Africa, Southeast Asia, the Middle East, Central/Eastern Europe, and Latin America. Data on the prevalence of *T. gondii* show different prevalence rates in Asia (13.3–85.3%), Europe (40–76%), Africa (21.74–74.8%), and North America (7.3–26.5%) [4].

In Bulgaria, studies using immunological methods to determine the prevalence of toxoplasmosis in different population groups have been carried out since the early 1960s to the present day. During this extended period, the examination of more than 800,000 individuals has been conducted, predominantly pregnant women and patients exhibiting symptoms such as lymphadenitis, eye inflammation, vague febrile state, and, since the 1990s, individuals living with HIV, with the objective of facilitating the early diagnosis of *Toxoplasma* infection. The average overall seropositivity rate for the period 1964 to 2023 was 29% [5,6,7,8,9,10,11,12,13,14,15,16,17]. Given that the diagnosis of toxoplasmosis is primarily conducted through the utilization of immunological methods, a substantial number of serological reactions have been consistently endorsed and employed within the country’s diagnostic framework. These include the Sabin–Feldman test, the complement fixation reaction, the passive hemagglutination reaction, and, after 1990, tests based on enzyme immunoassays. In the late 1990s and early 2000s, the study of individual antibody classes began to enable the stage of infection to be determined [10,11].

According to the prevailing legislation in Bulgaria, mandatory notification and registration are required only in the case of congenital toxoplasmosis. Consequently, information concerning the prevalence of toxoplasmosis in the country is often presented in a summarized form and may be incomplete, with the exception of data pertaining to congenital toxoplasmosis. In this context, the present study was initiated with the aim of studying the prevalence of this infection in the country over the last decade, with a particular focus on pregnant women, due to the psycho-emotional, family, and social impacts associated with the birth of a child with congenital disabilities and/or miscarriages caused by *T. gondii* infection.

## 2. Materials and Methods

### 2.1. Study Design

The present study was conducted at the Department of Parasitology and Tropical Medicine at the National Center for Infectious and Parasitic Diseases (NCIPD), Sofia, Bulgaria. It was a retrospective cross-sectional study covering the period from January 2014 to December 2023. With regard to the prevalence of toxoplasmosis at the national level, data from the annual reports of the Regional Health Inspectorates (RHIs) on diagnosed parasitic infections from the respective administrative regions of the country were used.

With regard to the prevalence of toxoplasmosis among pregnant women, data from the National Reference Laboratory for the Diagnosis of Parasitic Diseases (NRL) at NCIPD were utilized for this particular period. The laboratory performs a comprehensive array of serological tests, enabling the determination of the presence of anti-*Toxoplasma* specific IgG, IgM, and IgA antibodies, as well as the avidity values of specific IgG antibodies. Furthermore, real-time PCR for the detection of parasite DNA in clinical materials has been introduced into the laboratory’s diagnostic practice. Due to the increased diagnostic capabilities of the NRL, most patients suspected of toxoplasmosis across the country are referred to it for diagnostic clarification and therapeutic management. Patients tested for toxoplasmosis in the laboratory are both immunocompetent and immunocompromised with a variety of clinical symptoms, but the largest group of individuals tested are pregnant women

### 2.2. Data Collection and Study Population

In Bulgaria, the system for the surveillance of communicable diseases, including parasitic infections, facilitates the collection of annual data from all diagnostic laboratories nationwide. This information is collated by the NCIPD, specifically the Department of Parasitology and Tropical Medicine. The scope of these data encompasses the number of individuals examined in the given year, categorized by nosological entity, along with the proportion of these cases diagnosed with parasitic infections. The collection of this information is the responsibility of the Regional Health Inspectorates (RHIs) in the respective administrative regions of the country. Subsequent to the collection of this information, it is then sent to the NCIPD for processing and analysis in accordance with existing legislation.

With regard to the prevalence of toxoplasmosis in women of childbearing age, and, in particular, in pregnant women, we used data from the reference laboratory on immunocompetent women tested for toxoplasmosis without evidence of clinically manifest disease, which we divided into two groups.

The first group included pregnant women from all over the country who underwent a diagnosis of parasitic diseases at the NRL during the study period. Most were referred to the laboratory by their attending obstetrician, while others were tested on request as pet owners or because of contact with stray cats. According to the established diagnostic algorithm in the laboratory, different follow-up approaches are applied: (i) in the case of a negative result, health education is given to the expectant mother and follow-up examinations at 8–12 weeks are recommended in order to detect possible seroconversion over time during pregnancy; (ii) the presence of specific IgG antibodies and absence of IgM is considered to indicate latent infection (longer than the six-month risk period for vertical transmission); and (iii) the presence of specific *Toxoplasma* IgG and IgM antibodies is considered to be a probable indicator of a recently acquired infection. In these cases, because of the possibility of the persistence of specific IgM antibodies for more than one year and to clarify the time of infection, the level of *Toxoplasma* IgA is investigated. IgA kinetics in the context of toxoplasmosis have been shown to demonstrate that the level of these specific antibodies increases at around day 7 of infection and persists for approximately 6 months, after which it gradually declines to negative values [18]. In recent years, when acute infection is suspected, it has also become mandatory to test the avidity of *Toxoplasma* IgG antibodies to more accurately determine the stage of infection. All pregnant women diagnosed with acute toxoplasmosis were subsequently consulted by a parasitologist at the NCIPD and prescribed treatment, with follow-up and control examinations until delivery. The duration of drug treatment was determined individually according to the dynamics of their specific anti-*Toxoplasma* antibodies and IgG avidity values.

The second group included women of childbearing age from all over the country who were tested for toxoplasmosis because of an upcoming in vitro procedure or after an abortion for various reasons. Most were referred for testing by assisted reproduction centers or other health facilities.

### 2.3. Serum Samples

For serological testing, a blood sample obtained by venipuncture with a vacuum system was taken from patients admitted to the laboratory. After the separation of the serum, the samples were stored at −20 °C until testing.

### 2.4. Methods of Serological Diagnosis

The levels of specific *Toxoplasma* antibodies in the reference laboratory were tested with commercial test kits based on the enzyme-linked immunosorbent assay (ELISA) method. Serum samples were analyzed for the presence of specific *Toxoplasma* IgG, IgM and IgA a antibodies and/or IgG avidity. All tests were performed and interpreted according to the manufacturer’s instructions. A quantitative ELISA method (Platelia Toxo IgG, Bio-Rad, Paris, France) was used to detect specific *Toxoplasma* IgG levels. Values above 6 IU/mL were reported as positive. For specific IgM and IgA antibodies, the ELISA tests were of the semi-quantitative double-sandwich type (Platelia Toxo IgM and Platelia Toxo IgA, Bio-Rad, France). The presence of IgM and IgA antibodies was determined by comparing the optical density of serum samples with that of the cut-off of the reaction. When a serum ratio (SR) ≥ of 1 was obtained, the result was considered positive, between 0.8 and 1.0 as borderline, and ≤0.8 as negative.

If necessary, the avidity of *Toxoplasma gondii* IgG was determined (PlateliaToxo IgG Avidity, Bio-Rad, Paris, France). Results below 40% were interpreted as low, between 40% and 50% as borderline, and above 50% as high avidity.

### 2.5. Statistical Analysis

Data extraction was performed using a Microsoft Excel worksheet, and statistical processing of the data was conducted with GraphPad Prism 9 software. Descriptive indicators such as mean, median, and standard deviation were determined and Chi-square and Mann–Whitney tests were used to detect statistically significant differences in the studied indicators.

## 3. Results

### 3.1. Serological Testing for Toxoplasmosis in the Bulgarian Population

During the study period, a total of 115,053 persons were tested for clinical, epidemiological, and prophylactic indications in the country’s parasitological diagnostic laboratories. The average prevalence of toxoplasmosis for the period was 16.96%, and data by year are presented in Table 1.

### 3.2. Serological Tests for Toxoplasmosis in Pregnant Women

A total of 631 pregnant women aged between 19 and 47 years (mean age 31 ± 4.9 years) were examined in the NRL for the Diagnosis of Parasitic Diseases. There were 221 in the first trimester of pregnancy, 292 in the second, and 118 in the third trimester. All were initially tested for the presence of *Toxoplasma* IgG and IgM antibodies. If their levels were found to be above the reference for the diagnostic kit, the study was expanded to include testing for the presence of specific IgA and determination of the avidity of *Toxoplasma* IgG, according to the manufacturer’s instructions. Antibodies to *Toxoplasma gondii* were not detected in 320 women (50.7%). The remaining 311 pregnant women were initially found to have specific IgG antibodies, with an overall IgG seropositivity of 49.3% and a mean of 136.6 IU/mL ± 74.98 SD. Laboratory evidence of latent toxoplasmosis (presence of anti-*Toxoplasma* IgG antibodies only) was found in 156 patients (24.7%). IgM antibodies were present in 155 (24.5%) of the pregnant women, and specific IgA was detected in 50 (8%) of them (Table 2).

Demographic data show that the mean age of pregnant women in Bulgaria is 31 years, with the highest number in the 30–34 age group—232 (35.6%). The relative proportion of those seropositive for specific IgG, IgM, and IgA antibodies was highest in women aged 26–34 years. However, there was no statistically significant difference between seronegative pregnant women and those with specific antibodies to *T. gondii* by age group (*p* > 0.05) (Table 3).

#### 3.2.1. Quantitative Values of Specific IgG Antibodies

The mean value of *Toxoplasma* IgG antibodies in all 311 positive pregnant women was 136.6 IU/mL ± 74.98 SD. In the group of 156 women with evidence of latent toxoplasmosis and positive results only for the presence of specific IgG antibodies, the mean value was 112.1 IU/mL ± 66.75 SD. In pregnant women with data on recent infection (IgG+, IgM+, IgA− and IgG+, IgM+, IgA+), the mean value for IgG was 165.2 lU/mL ± 72.92 SD. There was a statistically significant difference between the mean IgG levels of pregnant women with latent infections and those with recently acquired infections (*p* < 0.00001).

#### 3.2.2. Testing for the Presence of *Toxoplasma* IgM and IgA Antibodies

Specific *Toxoplasma* IgM antibodies were detected in 155 pregnant women. The mean SR of specific IgM was 3.11 ± 1.65 SD in women without the presence of specific IgA (n = 105) and 4.2 ± 2.24 SD in those with the presence of specific IgA (n = 50). The difference in mean values was statistically significant (*p* = 0.0139).

#### 3.2.3. *Toxoplasma* IgG Avidity Test

This indicator, which can largely determine the stage of infection, was tested in 143 (92%) of the pregnant women with the presence of specific IgM antibodies (n = 155). Low and borderline avidity was found in 33 (23%) of those tested with the presence of specific IgG, IgM, and IgA antibodies (n = 50), and in 32 (30.5%) of those positive for IgG and IgM (n = 105). The comparison of low avidity values (up to 40%) in pregnant women with data for IgG and IgM only (mean 28%) and those with IgG, IgM and IgA antibodies (mean 24%) showed no statistically significant difference (*p* > 0.05). High avidity (more than 50%) was detected in 78 (54.5%) of the subjects, of which 6 pregnant women were positive for IgG, IgM, and IgA and 72 for IgG and IgM antibodies only. The results showed that current infection was detected in 66% (33 of 50) of the pregnant women with anti-*Toxoplasma* IgG, IgM, and IgA, and in 30.5% (32 of 105) of those with specific IgG and IgM antibodies, with a statistically significant difference (*p* < 0.05) between the two groups with evidence of recent infection.

#### 3.2.4. The Relationship Between the Stage of Infection and Gestation

In the first and second trimesters of pregnancy, data on the presence of IgG and IgM were obtained in 87 (83%) of pregnant women and in 44 (88%) of those with IgG, IgM, and IgA antibodies (Table 4). In the first trimester and with low and borderline avidity were 12 (11.4%) of pregnancies with specific IgG and IgM, and 9 (18%) of those with IgG, IgM, and IgA antibodies.

### 3.3. Serologic Testing for Toxoplasmosis in Women Applying for In Vitro Procedures or After Abortion

In the second group, a total of 161 women of childbearing age were screened for toxoplasmosis between 2014 and 2023, of whom 122 (75.8%) were screened for an in vitro procedure and 39 (24.2%) after an abortion. Their mean age was 35 ± 3.8 SD and, as with the pregnant women, the largest number were examined in the 30–34 age group (n = 62, 39.8%) (Table 5).

The ELISA testing of serum samples showed the presence of specific IgG antibodies in 72 (44.7%) of the subjects (n = 161), with a mean value of 126.4 IU/mL ± 80.4 SD (n = 58, 80.6% of women applying for an in vitro procedure; n = 14, 19.4% of women after an abortion). The study revealed that only *Toxoplasma* IgG antibodies were detected in a total of 45 (28%) individuals, with mean values of 120 IU/mL ± 81.2 SD (in vitro n = 35, 77.8%; after abortion n = 10, 22.2%). Furthermore, it was observed that women with specific IgG and IgM antibodies (n = 21) exhibited IgG values of 126 IU/mL ± 68.6 SD, while those with IgG, IgM, and IgA (n = 6, 3.7%) demonstrated IgG levels of 217 IU/mL ± 28.47 SD. A comparison of mean IgG levels in patients with evidence of latent and acute infection showed no statistically significant difference, despite higher IgG levels in those with IgG, IgM, and IgA antibodies (*p* = 0.18684).

*T. gondii* IgM antibodies were detected in 27 (16.8%) individuals, with a mean SR of 3.4 ± 1.9 SD (n = 23, 85% of women applying for in vitro fertilization; after abortion, n = 4, 15%). Comparison of the mean SR values of specific IgM antibodies in subjects with IgG and IgM only (n = 21) and those with IgG, IgM, and IgA antibodies (n = 6, SR 3.8 ± 2.7 SD) showed no statistically significant difference (*p* = 0.92034). *Toxoplasma* IgA was only detected in women applying for an in vitro procedure (Table 6).

The determination of IgG avidity in this group was performed in 22 (13.6%) of the tested women, and low and borderline values were found in 4 of them—2 with the presence of IgG and IgM and 2 with IgG, IgM, and IgA antibodies—all from the group being tested for an upcoming in vitro procedure.

## 4. Discussion

In the present study, conducted over a 10-year period, it was shown that nearly 17% of persons tested for toxoplasmosis in Bulgaria were positive for specific *Toxoplasma* IgG antibodies [14,15,16,17]. However, these are data from routine diagnostic work-ups performed for indications (clinical, epidemiological, or prophylactic) on patients suspected of having the infection, and not based on a focused scientific study aimed at determining seroprevalence for toxoplasmosis in the general population of the country. Therefore, the data regarding the study contingent of women of childbearing age (pregnant, with an upcoming in vitro procedure, or after an abortion), which is the focus of our study, are not very surprising.

For this period, we found an overall seropositivity for specific IgG antibodies in this cohort of 48.35%. In the two different groups studied, 631 pregnant women at various stages of pregnancy and 161 after an abortion or before an in vitro procedure, the seroprevalence was 49.3% (311 of 631) in the first group and 44.7% (72 of 161) in the second group. Latent infection was found in 156 (24.7%) of the pregnant women and in 45 (28%) of the women after an abortion or who were planning an in vitro procedure. Serological evidence of current infection was found in 155 (24.6%) of the pregnant women and in 27 (16.7%) of the women in the second group. Knowledge of the kinetics of specific antibodies in toxoplasmosis and the study of IgG avidity values refined the number of subjects in whom an acute form of toxoplasmosis and the possibility of congenital fetal infection could be determined with certainty. This was particularly true for women in the first trimester of pregnancy, whose relative proportion was 3.3% of the pregnant women included in the study. According to our management algorithm, all pregnant women with the presence of specific IgG, IgM, and/or IgA antibodies and low/border avidity, regardless of gestational age, are recommended to receive Spiramycin for the prevention of fetal infection and monthly serological monitoring. In the presence of evidence from AG monitoring and imaging of fetal developmental problems, amniocentesis is recommended after 4 months of gestation and testing for the possible presence of *Toxoplasma* DNA in the amniotic fluid. Despite the diagnostic options for toxoplasmosis available in the country, the lack of mandatory screening for pregnant women leads to the occurrence of congenital infection in some cases. Between 2017 and 2022, 10 cases of congenital toxoplasmosis were reported in the country, 2 of which were confirmed by real-time PCR at the reference laboratory after delivery.

Previous studies of the seroprevalence of *Toxoplasma* infection, including patients who underwent testing at the laboratory, showed similar results in pregnant women. For the period 2011-2020, 18.74% of 944 pregnant women examined were found to meet the criteria for acute toxoplasmosis, and 4.87% also showed low IgG avidity in the presence of specific IgM and/or IgA antibodies [19]. Another 2014–2016 study of 301 pregnant women demonstrated a positive result for *Toxoplasma* IgG in 49.2%. Of these, IgM antibodies were detected in 15.6% of the women, and specific IgA in 6.6%. The significant number of patients diagnosed with specific *Toxoplasma* IgG, IgM, and IgA, which does not show any particular dynamics over the years, is probably due to the fact that the reference laboratory is the only one in the country and that susceptible patients and those requiring consultation and the clarification of results from other laboratories are sent for testing [20].

The comparison of the data from our retrospective study with results from studies of pregnant women in Bulgaria’s neighboring countries shows significant differences in the seroprevalence of specific IgG and IgM in different countries and their regions. Typically, the seroprevalence of *Toxoplasma* infection is higher in southern and south-eastern European countries than in northern and western European countries [21]. Results from a 15-year (2008–2023) screening program for toxoplasmosis in pregnant women in Romania showed that the overall seroprevalence for specific IgG was 29.12% among 27,169 pregnant women tested. Acute *Toxoplasma* infection was suspected in 0.16% of those tested, due to seroconversion or low/border avidity [22]. Another study, which included 2626 women of childbearing age from the Arad district in 2016–2018, reported 41.16% seropositivity for *Toxoplasma gondii* [23]. In Belgrade, Serbia, a cross-sectional study of 300 healthy pregnant women in late 2018 and early 2019 showed a seroprevalence of 12.7% for *Toxoplasma* IgG antibodies [24]. No pregnant women were identified with evidence of specific IgM antibodies. However, in a study of 765 women of reproductive age from all over Serbia between 2001 and 2005, the prevalence of this parasitosis was 33% [25].

In a study conducted in northern Greece for 1984, 1994, and 2004, gradual decreases in IgG seropositivity to *T. gondii* was observed in women of reproductive age (15–39 years) of 35.6%, 25.6% and 20%, respectively. The authors point out that between 90 and 200 newborns are infected within the uterus each year, representing a potential public health risk, and that education and prophylactic measures to prevent congenital toxoplasmosis are becoming increasingly important [26]. On the island of Crete, *Toxoplasma* infection was monitored during 1998–2003 in 5532 pregnant women, with a seroprevalence of 29.45% and 3.34% fulfilling the criteria for acute toxoplasmosis [27].

Data from studies of seroprevalence in pregnant women in Turkey over the past 30 years have been reported in a number of publications, in which the authors found that the mean relative proportion of pregnant women with a presence of *Toxoplasma* IgG was 36.76%, and the presence of specific IgM was found in 2.91% of them [28].

In a systematic review and meta-analysis of studies conducted between 1993 and 2023, the pooled seropositivity rate for toxoplasmosis was 32.3% in the Spanish population, but 24.4% in pregnant women [29]. Data from surveys in areas of central (Siena) and southern (Bari) Italy show that the percentage of seropositive patients in Bari is significantly higher than in Siena (22.4% vs. 12.4%), and that there is an age-related trend [30]. In northern Italy (province of Bologna, region of Emilia Romagna), the screening of a population of 36,814 individuals revealed a prevalence of toxoplasmosis of 20.4% and a prevalence of active infection of 0.39% in pregnant women [31].

In France, where a screening program for toxoplasmosis has been in place since the 1970s, perinatal screening conducted in 2016 showed seroprevalence data for *T. gondii* in pregnant women of 31.3%, a significant decrease compared to 1995 when it was 54% [32].

The age range of the pregnant women included in our study was 19–47 years (mean age 31 ± 4.9 years). Although almost half (46.5%) of the pregnant women with a positive serological result for toxoplasmosis were aged 25–34 years, there was no statistically significant association between age and *T gondii* infection. Similar results have been reported by authors from Serbia and Italy, while in Romania the proportion of *Toxoplasma* IgG-positive women increased with age and the differences in prevalence between age groups were statistically significant [22,24,33]. In Bulgaria, Romania, Serbia, and France, the mean age of pregnant women is increasing and is associated with older age groups between 30 and 39 years [22,24,32].

Studies of risk factors for *T. gondii* infection in humans in Bulgaria are episodic. In 2022, researchers from the Stara Zagora region (southern Bulgaria) reported five variables positively associated with toxoplasmosis (*p* < 0.05), including urban residence, gardening or farming, the consumption of undercooked meat, contact with yard cats, and knowledge of toxoplasmosis as a disease. The authors tested 365 individuals aged between 7 and 72 years (mean age of seropositivity, 32.3 ± 11.8 years) and found a seroprevalence for specific IgG of 47.9% [34]. In our previous study of prevalence against *T. gondii* among pregnant women for the period 2014-2016, the available data led us to conclude that the main risk factors for infection were more associated with the consumption of poorly washed fruits and vegetables [20].

The consumption of raw vegetables is also associated with toxoplasmosis in other countries, such as France and Slovakia [35,36]. Despite some national specificities, the main risk factors for *T. gondii* infection described in different studies are similar [21,22,24,35].

Despite the important epidemiological role of cats in the spread of toxoplasmosis, studies on their infection with oocysts or tissue cysts are scarce in Bulgaria. The seroprevalence of toxoplasmosis in street and domestic cats has been studied in Plovdiv (southern Bulgaria) for the period 2018–2022. A total of 56 serum samples from cats (30 stray and 26 domestic) were tested, and antibodies to *T.gondii* were found in 36.6% of stray and 7.7% of domestic cats [37]. A link between stray cats and the risk of spreading toxoplasmosis was also found by authors from the Czech Republic [38]. In Bulgaria, the lack of official statistics does not allow an exact determination of the number of cats, but data from the annual game control carried out by the Union of Hunters and Fishermen in Bulgaria show that for the period 2017–2019 the number of stray cats in the country was almost 12,000 [39].

A possible route of infection is also the consumption of undercooked meat or meat products. In a study of 380 sheep and 364 goats from different villages and farms, the presence of specific IgG antibodies was found in 48.2% of sheep and 59.8% of goats, and IgM was found in 6.7% (5 of 75) and 6% (11 of 179), respectively [40]. The spread of toxoplasmosis among animal hosts could have a significant impact on its spread among humans, although no data on the consumption of undercooked meat and meat products were found in previous studies of pregnant women [20]. In recent years, the risk of infection with *T. gondii* has been associated more with the consumption of chicken than lamb or other meats [41]. In Bulgaria, there are no studies and therefore no data on the parasite burden in chickens or chicken meat but, nevertheless, poultry meat should be properly prepared as a preventive measure.

The studies conducted so far in Bulgaria on the seroprevalence of toxoplasmosis in pregnant women have not found evidence of a higher infection rate among those living in rural areas. On the contrary, seropositivity is higher among pregnant urban women. This may be explained by the fact that in Bulgaria the relationship between town and village is not sharply demarcated, as most people living in towns have close ties with villages through parents, visits to relatives, or farms [42]. Regarding this risk factor, the data from our study differ from the data from Romania, where a higher seroprevalence of specific IgG antibodies was found in pregnant women living in rural than in urban areas [22]. Similar data have been presented by other authors, according to which seropositivity of *T. gondii* is increasing in rural populations [21].

The other group of women studied (n = 161) were those after miscarriage or abortion for medical reasons (n = 39) or applying for an in vitro procedure (n = 122). The seropositivity was 25.6% for specific IgG and 10% for IgM in the women after an abortion. Due to their high avidity values, we can assume that there is no evidence of recent infection. However, other studies with similar seropositivity for *Toxoplasma* IgG and IgM in women after abortion have suggested that *Toxoplasma* infection may be a possible cause of miscarriage [43,44]. In this sense, we strongly support the view that in such cases, or in conditions predisposing to such an outcome, toxoplasmosis must necessarily be included in the differential diagnostic plan as a possible cause.

With regard to screening for toxoplasmosis prior to in vitro procedures, it could be said that such screening is necessary and useful, as it avoids a number of medical and psycho-emotional problems associated with pregnancy and the risk of intrauterine infection of the fetus.

## 5. Conclusions

The seroprevalence of *Toxoplasma* IgG and IgM antibodies in pregnant women, post-abortive women, and those planning in vitro procedures was higher than in neighboring countries. A study involving a larger number of pregnant women throughout the country is needed to clarify the infectivity of *T. gondii* and the possibility of congenital infection. This could be performed by introducing screening programs for diseases that pose a risk to pregnancy and the fetus, including toxoplasmosis. There is also a need to test for oocyst infestation of soil in parks and gardens, as well as yards around houses. In Bulgaria, about 10,000 people are tested for toxoplasmosis annually, so there is an opportunity to increase the population’s knowledge of the infection, its transmission mechanisms, and prevention measures.

## Figures and Tables

**Table 1:** Number of individuals examined and seroprevalence for toxoplasmosis for 2014–2023 (determination of seroprevalence based on presence of specific IgG).

Year	Number of Examined Individuals	Number of Individuals with Presence of Specific Anti-*Toxoplasma* IgG Antibodies	Prevalence
**2014**	13,545	2177	16.07
**2015**	10,233	2108	20.6
**2016**	10,041	1976	19.68
**2017**	10,525	2128	20.23
**2018**	11,648	2086	17.91
**2019**	14,242	2588	18.17
**2020**	9521	1464	15.37
**2021**	10,203	1497	14.67
**2022**	8773	1455	16.58
**2023**	16,322	1680	10.29
**Total**	115,053	19,159	16.96

**Table 2:** Serological status of examined pregnant women.

Serological Status	Pregnant Women (n)	Relative Proportion (%)	Median Value of IgG (IU/mL)	Median Value of IgM (SR)	Median Value of IgA (SR)	Interpretation
**IgG (−), IgM (−)** **IgA (−)**	320	50.7	(−)	(−)	(−)	Absence of infection
**IgG (+), IgM (−), IgA (−)**	156	24.7	112.1 IU/mL ± 66.75 SD	(−)	(−)	Latent *Toxoplasma* infection
**IgG (+), IgM (+), IgA (−)**	105	16.6	165.2 lU/mL ± 72.92 SD	3.11 ± 1.65 SD	(−)	Acute *Toxoplasma* infection
**IgG (+), IgM (+), IgA (+)**	50	8	165.2 lU/mL ± 72.92 SD	4.2 ± 2.24 SD	2.04 ± 1.79 SD	Acute *Toxoplasma* infection
**Total**	631	100				

**Table 3:** Demographic characteristics according to age group of pregnant women.

Age Group	Total n = 631 (%)	*T. gondii* Negative n = 320 (%)	IgG Positive n = 156 (%)	IgM and IgM + IgA Positive n = 155 (%)	*p*-Value
**15–19**	2 (0.3)	1 (0.3)	0	1 (0.6)	
**20–24**	53 (10.1)	20 (6.3)	11 (7)	22 (14.2)	0.914
**25–29**	198 (32)	110 (34.4)	45 (28.8)	43 (27.7)	0.962
**30–34**	232 (35.6)	120 (37.5)	58 (37.2)	54 (34.8)	0.938
**35–39**	119(18.1)	56 (17.5)	34 (22)	29 (18.7)	0.944
**>40**	27 (3.9)	13 (4)	8 (5)	6 (4)	0.847

**Table 4:** Tests results for *Toxoplasma* IgG, IgM, and IgA antibodies and data on IgG avidity during pregnancy.

Status of Toxoplasmosis	*T. gondii* Seropositive Pregnant Women	First Trimester n (%)	Second Trimester n (%)	Third Trimester n (%)
**Acute infection**	IgG (+), IgM (+), IgA (+); low/borderline IgG avidity	9 (18)	18 (36)	3 (6)
	IgG (+), IgM (+), IgA (−); low/borderline IgG avidity	12 (11.5)	10 (9.5)	8 (7.6)
**Latent infection**	IgG (+), IgM (−)	50 (32)	76 (49)	30 (19)

**Table 5:** Demographic characteristics of women tested for toxoplasmosis applying for in vitro procedure or after abortion.

Age Group	Total n = 161 (%)	*T. gondii* Negative n = 89 (%)	IgG Positive n = 45 (%)	IgG, IgM and IgG, IgM, IgA Positive n = 27 (%)	*p*-Value
**20–24**	4 (2.4)	1 (1.1)	1 (2.22)	2 (7.4)	0.776
**25–29**	26 (16.8)	16 (18)	8 (17.8)	2 (7.4)	0.996
**30–34**	62 (39.8)	37 (41.6)	14 (31.1)	12 (44.4)	0.520
**35–39**	38 (22.4)	19 (21.3)	11 (24.44)	7 (26)	0.767
**>40**	31 (18.6)	16 (18)	11 (24.44)	4 (14.8)	0.807

**Table 6:** The serological status of women with an imminent in vitro procedure or after an abortion.

Serological Status	Women for In Vitro (n, %)	Women after Abortion (n, %)	Median Value of IgG (IU/mL)	Median Value of IgM (SR)	Median Value of IgA (SR)	Interpretation
**IgG (−), IgM (−)** **IgA (−)**	64	25	(−)	(−)	(−)	Absence of infection
**IgG (+), IgM (−), IgA (−)**	35	10	120 IU/mL ± 81.15 SD	(−)	(−)	Latent *Toxoplasma* infection
**IgG (+), IgM (+), IgA (−)**	17	4	126 IU/mL ± 68.6 SD	3.4 ± 1.9 SD	(−)	Acute *Toxoplasma* infection
**IgG (+), IgM (+), IgA (+)**	6	0	217 IU/mL ± 28.47 SD	3.8 ± 2.7 SD	2.7 ± 2.3 SD	Acute *Toxoplasma* infection
**Total**	122	39				

## Data Availability

The data that support the findings of this study are available from the corresponding author upon reasonable request.
